# Identification of *MYH6* as the potential gene for human ischaemic cardiomyopathy

**DOI:** 10.1111/jcmm.17015

**Published:** 2021-10-26

**Authors:** Jian‐Hong Chen, Lei‐Li Wang, Lin Tao, Bin Qi, Yong Wang, Yu‐Jie Guo, Liu Miao

**Affiliations:** ^1^ Department of Cardiology Liuzhou People’s Hospital Liuzhou China; ^2^ Department of Oncology Liuzhou People’s Hospital Liuzhou China

**Keywords:** functional enrichment, functional validation and prognostic analysis, gene expression omnibus, ischaemic cardiomyopathy

## Abstract

The present study aimed to explore the potential hub genes and pathways of ischaemic cardiomyopathy (ICM) and to investigate the possible associated mechanisms. Two microarray data sets (GSE5406 and GSE57338) were downloaded from the Gene Expression Omnibus (GEO) database. The limma package was used to analyse the differentially expressed genes (DEGs). Kyoto Encyclopedia of Genes and Genomes (KEGG) pathway enrichment, Disease Ontology (DO) and Gene Ontology (GO) annotation analyses were performed. A protein‐protein interaction (PPI) network was set up using Cytoscape software. Significant modules and hub genes were identified by the Molecular Complex Detection (MCODE) app. Then, further functional validation of hub genes in other microarrays and survival analysis were performed to judge the prognosis. A total of 1065 genes were matched, with an adjusted *p* < 0.05, and 17 were upregulated and 25 were downregulated with|log_2_ (fold change)|≥1.2. After removing the lengthy entries, GO identified 12 items, and 8 pathways were enriched at adjusted *p* < 0.05 (false discovery rate, FDR set at <0.05). Three modules with a score >8 after MCODE analysis and MYH6 were ultimately identified. When validated in GSE23561, *MYH6* expression was lower in patients with CAD than in healthy controls (*p* < 0.05). GSE60993 data suggested that *MYH6* expression was also lower in AMI patients (*p* < 0.05). In the GSE59867 data set, *MYH6* expression was lower in CAD patients than in AMI patients and lower in heart failure (HF) patients than in non‐HF patients. However, there was no difference at different periods within half a year, and HF was increased when *MYH6* expression was low (*p* < 0.05–0.01). We performed an integrated analysis and validation and found that *MYH6* expression was closely related to ICM and HF. However, whether this marker can be used as a predictor in blood samples needs further experimental verification.

## 1 INTRODUCTION

Ischaemic cardiomyopathy (ICM) refers to the failure of the heart to pump blood normally due to myocardial damage caused by ischemia, and ICM is the leading cause of death globally according to the WHO. In addition, ICM is also the common cause of heart failure (HF) in the developed world.^1^ Coronary artery disease (CAD) is one of the most common ischaemic cardiomyopathy diseases and is caused by coronary artery stenosis and myocardial insufficiency.^2^ Compared with a nonischaemic aetiology, heart failure secondary to ICM has been shown to be independently associated with mortality.^3^ In view of the high mortality rates caused by CAD and HF, the prevention and timely treatment of ICM are particularly important.^4^

With the development of bioinformatics analysis and high‐throughput sequencing technology, many sequencing data have provided notable results to identify the hub genes, interaction networks and pathways of ICM. As a complex multifactorial disease, ICM can be caused by genetic and environmental factors and their interactions, and many previous studies have been conducted on ICM from different aspects, especially at the genome level.^5^ However, most of these analyses have focused on a certain aspect of ischaemic cardiomyopathy, for instance, several studies have paid more attention to the aetiologic aspect, and others have focused on the pathogenic aspect, which lacks systematic analysis.^6^

In this way, to investigate the molecular mechanisms of ICM pathogenesis in depth, we first downloaded expression profile data related to CAD and acute myocardial infarction (AMI) from the Gene Expression Omnibus (GEO), and after systematic analysis, we selected potential genes for CAD pathogenesis. Then, we performed validation in several different data sets to further screen out the hub genes to identify the mechanisms of these hub genes in the pathogenesis of AMI. Finally, we validated the expression levels of these hub genes in additional datasets in relation to the onset of HF after AMI to identify the causative genes for ICM.

## 2 MATERIALS AND METHODS

### 2.1 Microarray data sets

A total of five microarray datasets were downloaded from the Gene Expression Omnibus (GEO) database (https://www.ncbi.nlm.nih.gov/geo/). The five microarray datasets were GSE5406,^7^ GSE57338,^8^ GSE23561,^9^ GSE60993 ^10^ and GSE59867,^11^ among which GSE5406 and GSE57338 were mainly used for data analysis to identify hub genes related to ICM, while the other datasets were used for validation. GSE5406 was retrieved from the GPL96 Affymetrix Human Genome U133A Array. In this data set, totally 124 subjects (including 108 related to ICM and 16 controls) were analysed. GSE57338 was retrieved from the GPL11532 Affymetrix Human Gene 1.1 ST Array. A total of 231 subjects (including 95 related to ICM and 136 controls) were chosen for further analysis. Samples of both of the above datasets were derived from heart tissue. The Affy package in R^12^ was used to transform CEL files into an expression value matrix, and the matrix was normalized using the RMA method. Then, we converted the probe data to genes with the Bioconductor package in R software.^13^ When multiple probes corresponded to a gene, the Arithmetic mean expression value of the probe was chosen for further analysis. GSE23561, GSE60993 and GSE59867 were used as the datasets set for validation, and the analysis methods were the same as those above.

### 2.2 **Differentially expressed genes (DEGs) and functional enrichment analysis**.

We compared ICM subjects with healthy controls to identify the differentially expressed genes (DEGs) with the limma package in R.^14^ Then, we set|log_2_ (fold change)|≥1.2 and adjusted *p* < 0.05 as the threshold for DEGs. Subsequently, we employed DOSE^15^ and the clusterProfiler^16^ package in R to perform the Kyoto Encyclopedia of Genes and Genomes (KEGG) pathway, Disease Ontology (DO) and Gene Ontology (GO) analyses for DEGs. An adjusted *p*‐value (Q‐value) of <0.05 was regarded as statistically significant. In addition, in our analysis, we focused our attention on hits with Q < 0.05 and, to avoid very general sets, limited our final list of hits to examine pathway sets that annotated fewer than 200 genes.

### 2.3 Protein‐protein interaction analysis (PPI) network construction and module analysis

The STRING database (version 11.0)^17^ is used to detect predicted and experimental interactions in a protein database, and we employed it to identify and predict protein‐protein interactions for the DEGs in this study. The database uses several prediction methods, such as cooccurrence, gene fusion, coexpression experiments, databases, neighbourhoods and text mining. In addition, the protein pair interactions are shown with the combined fraction in the database. In our current study, a combined score >0.9^18^ was set as a cut‐off value. We used degrees to reveal the roles of protein nodes in the network. We defined the key protein networks as network modules and speculated that they may have a specific biological effect in previous studies. Subsequently, Cytoscape (version 3.71) software and the Molecular Complex Detection (MCODE) app^19^, ^20^ were employed to detect the major and most notable clustering modules. For further subsequent analysis, we set EASE ≤0.05 and count ≥2 as the cut‐off value and MCODE score >8 as the threshold.

### 2.4 Validation of interest and survival analysis

Initially, we wanted to further understand the relationships between hub genes and several diseases related to ICM and downloaded GSE23561 for the training set. Next, we compared the relative expression levels of hub genes between healthy subjects and patients with acute coronary syndrome in GSE60993. Subsequently, in GSE59867, we compared the relative expression levels of hub genes in patients with CAD and acute myocardial infarction (AMI) and AMI patients at different time points who suffered from heart failure. We used the ggplot2 package^21^ to compare the expression differences, and the ‘survival’ package^22^ in R was used to perform overall survival (heart failure) and disease‐free survival analyses. Patients were divided into two groups (high vs. low) based on the hub gene expression level in comparison with the mean expression level of that hub gene. A Kaplan‐Meier survival plot was also constructed.

## 3 RESULTS

### 3.1 Data preprocessing

Before analysing GSE5406 and GSE57338, we first judged the quality of these two samples. Figure S1 shows that after quality control, all of the samples were well normalized. We obtained 54,560 expression probes separately from each gene expression profile, and the expression matrices of 19,537 genes were obtained from GSE5406, while 18,334 genes were obtained from GSE57338.

### 3.2 Identification of differentially expressed genes and functional annotation

**FIGURE 1:**
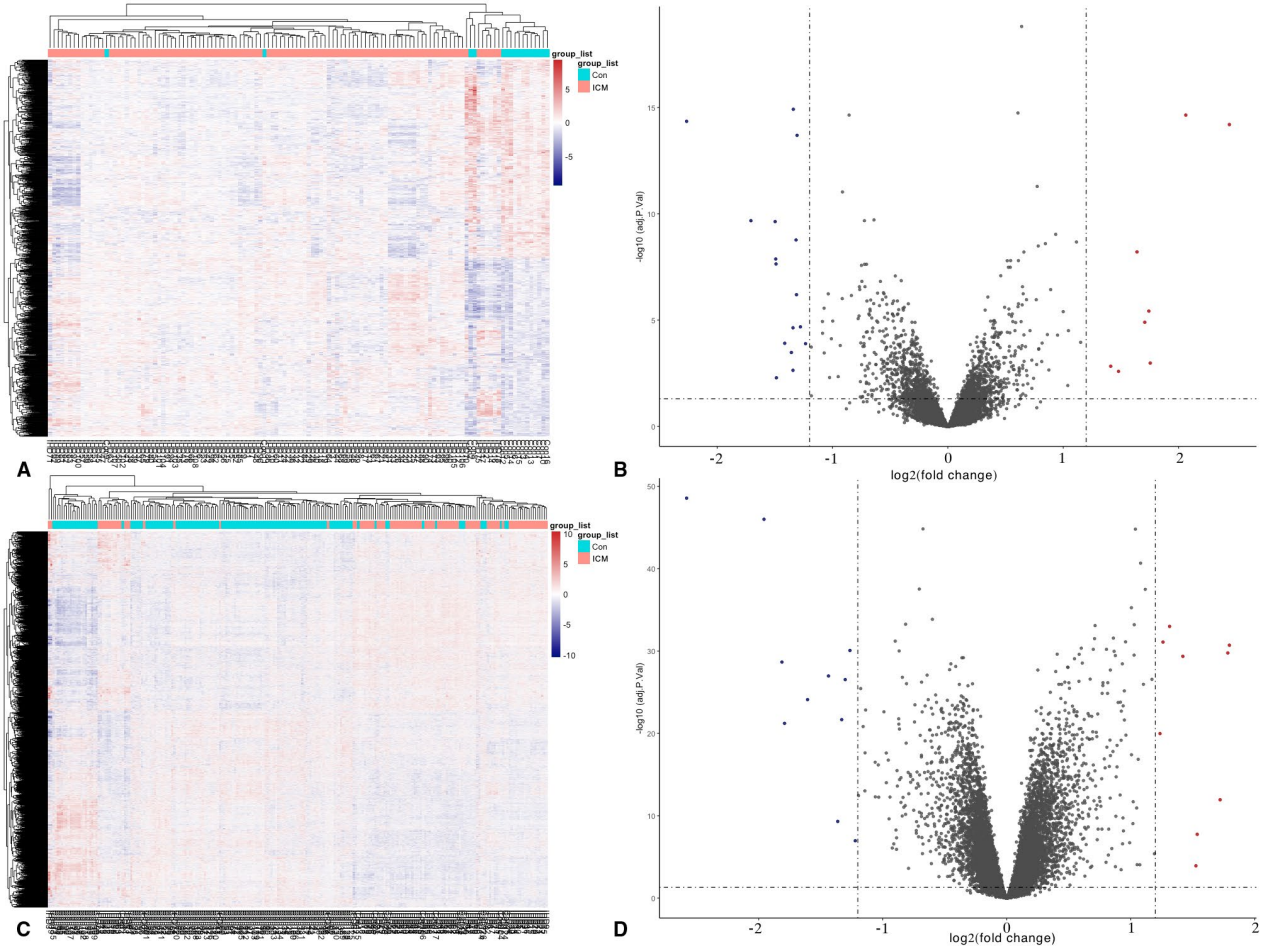
Heatmaps and volcano plots for DEGs. (A) Heatmap for DEGs in GSE5406; (C) Heatmap for DEGs in GSE57338. ICM groups are in the red cluster, and healthy samples are in the green cluster. (B) Volcano plot for DEGs in GSE5406; (D) Volcano plot for DEGs in GSE57338. The two vertical lines are the 1.2‐fold change boundaries, and the horizontal line is the statistical significance boundary (adjusted *p* < 0.05). Items with statistical significance and upregulation are marked with red dots, and downregulated items are marked with dark blue dots in the volcano plots

Upon comparing the case and control samples, we obtained a total of 1326 items with adjusted *p* < 0.05, though only 8 were upregulated and 7 were downregulated with|log_2_ (fold change)|≥1.2 in GSE5406, while among 8922 items, 9 were upregulated and 11 were downregulated in GSE57338. All of these DEGs are shown in Table 1, and the heatmaps and volcano plots are shown in Figure 1.

**TABLE 1:** Differentially Expressed Genes of these two datasets

SYMBOL	LogFC	adj.P. Val
GSE5406		
ASPN	2.44172693	6.39E−15
LUM	2.06368426	2.29E−15
EIF1AY	1.7554501	0.00105047
NPPA	1.74314047	3.78E−06
HBB	1.70770258	1.26E−05
MXRA5	1.64065971	6.23E−09
COL1A1	1.48037944	0.00258165
RPS4Y1	1.41257123	0.00148182
PLA2G2A	−1.234348	0.00012856
CYP4B1	−1.2789909	2.08E−05
SERPINA3	−1.3080511	2.06E−14
CCT2	−1.3130663	6.41E−07
CNN1	−1.3170948	1.71E−09
FCN3	−1.3405069	1.22E−15
ANKRD2	−1.3433277	2.30E−05
PTX3	−1.3435912	0.00230507
CD163	−1.3572422	0.00033415
MYH6	−1.4151743	0.00012331
NRAP	−1.4874166	0.00517698
GLUL	−1.49067	2.30E−08
FKBP5	−1.4934123	1.34E−08
HOPX	−1.4982989	2.34E−10
IL1RL1	−1.7082588	2.12E−10
MYOT	−2.2660804	4.51E−15
GSE57338		
SFRP4	1.7963568	1.96E−31
ASPN	1.7838173	1.70E−30
HBB	1.72188009	1.15E−12
NPPA	1.53711203	1.82E−08
EIF1AY	1.5265854	0.00012031
OGN	1.42120733	4.41E−30
FRZB	1.31416894	1.02E−33
LUM	1.2606985	8.12E−32
MXRA5	1.23699123	1.05E−20
HMGCS2	−1.2208725	1.09E−07
SLCO4A1	−1.2635669	8.55E−31
VSIG4	−1.3018988	2.94E−27
LYVE1	−1.3300046	2.14E−22
SERPINE1	−1.3620594	4.90E−10
CD163	−1.4366658	1.07E−27
MYH6	−1.6054559	7.95E−25
PLA2G2A	−1.7911045	6.06E−22
IL1RL1	−1.812301	2.17E−29
FCN3	−1.9567163	1.00E−46
SERPINA3	−2.5808474	2.70E−49

Note

*LogFC*, log_2_fold‐change; *adj*.*P*. *Val*, adjusted P value.

**FIGURE 2:**
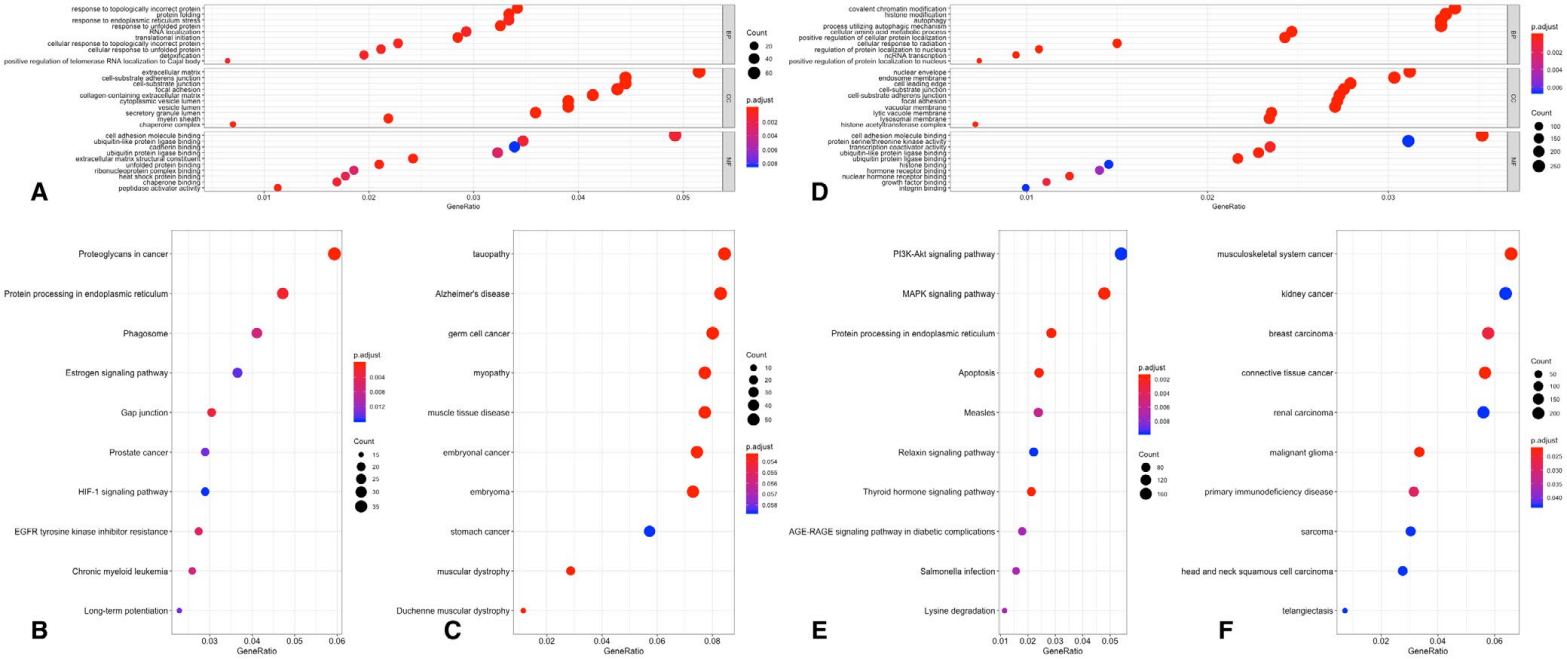
Functional enrichment analyses of DEGs. The x‐axis shows the ratio of the number of genes, and the y‐axis shows the pathway terms. The ‐log10 (*p*‐value) of each term is coloured according to the legend. (A) Gene Ontology in GSE5406; (B) Kyoto Encyclopedia of Genes and Genomes (KEGG) pathway analysis in GSE5406; (C) Disease Ontology in GSE5406; (D) Gene Ontology in GSE57338; (E) Kyoto Encyclopedia of Genes and Genomes (KEGG) pathway analysis in GSE57338; (F) Disease Ontology in GSE57338

Subsequently, we employed clusterProfiler and the DOSE package in R to carry out KEGG pathway enrichment, DO functional and GO analyses to elucidate the roles of the DEGs. In GSE5406, after the analysis of GO functions, totally 224 biological processes, 72 cellular components and 13 molecular functions were identified; 24 pathways were enriched for the KEGG pathway, and no DO items with an adjusted *p* < 0.05 were identified (Figure 2 TA‐C). Totally 825 biological processes, 157 cellular components and 46 molecular functions for GO functions were identified; 61 pathways were enriched for the KEGG pathway and 21 DO items with an adjusted *p* < 0.05 were identified in GSE57338 (Figure 2D–F). The details of these items are provided in Tables S1, 2 and 3.

**FIGURE 3:**
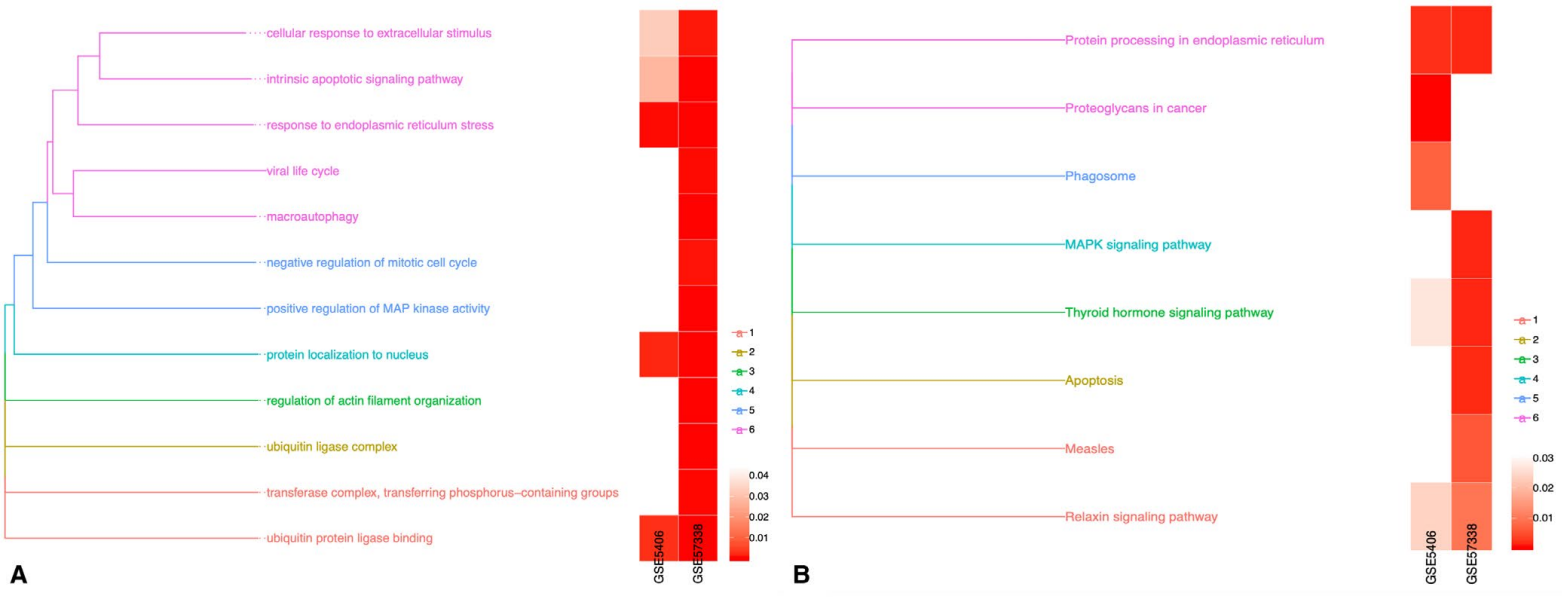
Removal of redundant entries for GO and KEGG analysis. GO and pathway categories were grouped according to functional themes, and the proportion of cases affected by individual pathway alterations was plotted per subgroup and across the series. (A) Gene Ontology and (B) Kyoto Encyclopedia of Genes and Genomes (KEGG) pathway analyses

Due to the complexity and verbosity of the above projects, many of them have similar content, so similar contents were merged (Figure 3). Among these items, GO:0043406 positive regulation of MAP kinase activity, hsa04141 protein processing in the endoplasmic reticulum, hsa04926 relaxin signalling pathway, hsa04919 thyroid hormone signalling pathway, hsa04010 MAPK signalling pathway and hsa05010 Alzheimer's disease was related to ICM. The genes related to these items were selected for further analysis. The details of these items of KEGG analysis can be found in Table 2.

**TABLE 2:** Kyoto Encyclopedia of Genes and Genomes analysis after simplify for two data sets

ID	Description	*P*. adjust	geneID
GSE5406			
hsa04141	Protein processing in endoplasmic reticulum	0.00210155	HSP90AA1/ATF4/SSR3/DERL1/DNAJB1/SEC61A1/UBE2D3/EIF2AK3/HSPH1/DNAJC3/CRYAB/BAG2/HYOU1/CUL1/SEC61G/UBE2G2/HSP90AB1/OS9/SEC23A/SEC31A/HSP90B1/HSPA2/MAN1C1/WFS1/XBP1/HSPA6/DERL2/DNAJB2/PLAA/DNAJA1/SSR1
hsa04926	Relaxin signalling pathway	0.02385391	MAP2K1/ATF4/COL1A2/PRKACB/NOS3/EDNRB/COL1A1/GNB5/CREB3/MMP2/TGFBR2/PIK3R3/COL3A1/GNB1/SHC1/MAPK1/PLCB4/ADCY1/SOS1/PLCB2/COL4A5/RAF1
hsa04919	Thyroid hormone signalling pathway	0.02688745	ATP1A1/MAP2K1/PRKACB/MYH6/MYC/ATP2A2/SLC16A2/RXRB/ESR1/PLCE1/PIK3R3/RCAN1/NOTCH2/MAPK1/PLCB4/ATP1B3/PLCG2/PLCB2/MTOR/RAF1
GSE57338			
hsa04010	MAPK signalling pathway	0.00153409	ELK1/MAP3K6/NF1/PPP3CC/MAP2K1/RPS6KA2/MAPKAPK2/ARAF/PLA2G4C/KRAS/DUSP7/IRAK1/CACNB1/MAPK10/CHUK/FGF1/PDGFC/PDGFRB/KIT/CACNA2D2/RAF1/CACNA1H/JUN/TGFBR2/RELA/CD14/SRF/ATF2/RAP1A/TNFRSF1A/TAB2/IL1R1/HSPA2/RPS6KA4/TAOK1/PDGFD/NGFR/CACNA2D1/CACNB4/NGF/MAPK8/MYD88/GNA12/NTRK2/PRKACB/RASA1/KDR/TEK/GADD45A/DUSP16/MKNK2/CDC25B/EFNA4/FLNC/FGF7/VEGFC/MAP3K7/AREG/IRAK4/IKBKG/RASGRP1/HRAS/DAXX/ERBB2/FGFR4/MAP2K2/MAP3K20/RASGRP2/RPS6KA1/PAK1/MAP4K3/RAPGEF2/PGF/MAP2K5/MEF2C/MAP3K14/FLT1/EPHA2/VEGFA/MYC/TGFB1/MAPK7/ARRB1/CRKL/FGF5/NTF3/ARRB2/GADD45B/GRB2/ERBB3/RASA2/RAC1/PDGFB/ECSIT/EFNA1/FLT4/ANGPT1/DUSP6/TAOK2/DDIT3/HSPB1/CSF1/SOS1/MAX/MRAS/PAK2/MAP3K3/TGFB2/CACNA1E/FGFR2/LAMTOR3/MAP2K7/MAP3K13/STK3/PRKCA/RPS6KA3/IGF1R/RPS6KA6/ELK4/GNG12/AKT1/FGF9/MAPK1/EREG/PPP5C/TP53/PPP3R1/FGF10/FGF20/MET/IGF1/FLT3LG/EFNA2/MAPT/ERBB4/MAP3K11/MAP3K4/PLA2G4F/MAPK14/PLA2G4E/EFNA3/ATF4/RELB/FGF18/CACNG3/CACNA2D3/RASGRP4/CACNA2D4/IL1B/CSF1R/PPM1B/PDGFA/PTPN5/ANGPT2/VEGFB/MAPK12/MAP3K8/FOS/CDC42/MECOM/PPP3CA/PPM1A/TRAF6/RASGRF2/HSPA1L/RASGRF1/PRKACG/CASP3/MAPK9/MAPK8IP1/FLT3
hsa04919	Thyroid hormone signalling pathway	0.00153409	CCND1/MYH6/ATP1A1/BMP4/MAP2K1/PLCE1/HIF1A/KRAS/DIO2/NCOA3/PLCB4/SLC2A1/ATP1B2/ATP1B3/MED12/RAF1/MED14/NCOA2/RXRG/RXRA/PIK3CB/PLCB1/PRKACB/PLCD3/ATP1B4/MED12L/ACTB/MED17/HRAS/ATP1B1/CTNNB1/ATP2A2/PIK3R3/RXRB/STAT1/MAP2K2/SLC16A2/PLCG2/RHEB/SRC/PIK3R1/PLCD4/HDAC3/PLCZ1/MYC/RCAN1/MED16/MTOR/EP300/NOTCH2/NOTCH1/ITGB3/PFKFB2/PLCB3/KAT2A/MDM2/NCOA1/PIK3CA/PRKCA/BAD/ESR1/AKT1/MAPK1/ATP1A3/TP53/THRB/ATP1A4/ACTG1/MED13/PLCG1/PLN/PRKACG/MED13L/RCAN2/MED1/PLCD1
hsa04141	Protein processing in endoplasmic reticulum	0.00160446	SSR3/NGLY1/CAPN1/UBQLN4/EIF2AK2/MAPK10/EDEM2/SEC61A1/CASP12/DNAJB12/UBE2J1/DERL1/RPN1/HSPA2/PPP1R15A/MAPK8/ATXN3/SEC23A/NPLOC4/DNAJC1/CRYAB/HYOU1/BAG2/ATF6B/UBE2J2/SEC24B/RAD23A/SEC62/BAX/AMFR/UBXN6/DNAJC5/HSPA4L/UBE2D2/BAG1/OS9/SEC31B/PRKN/ERLEC1/SVIP/PRKCSH/BAK1/MOGS/SEC24C/SELENOS/SEC61A2/DNAJB11/DNAJA2/EDEM1/SYVN1/UBE2D1/HSP90AA1/SEC13/STUB1/EIF2S1/MAN1B1/SEC23B/YOD1/DDIT3/ERO1A/ERO1B/UFD1/SAR1A/MAN1C1/XBP1/ATF6/UGGT1/NFE2L2/SEC61B/LMAN1/MAP2K7/PDIA4/SEC24A/EDEM3/NSFL1C/UBE2D3/UBQLN2/P4HB/SEC63/MBTPS2/HSPBP1/SEC61G/SKP1/HERPUD1/WFS1/SEL1L/HSP90AB1/ERN1/MARCH6/ATF4/PREB/SEC24D/LMAN2/UBE2G2/DERL2/HSPA1L/UBQLN1/EIF2AK4/BCAP31/CUL1/MAPK9/ERP29
hsa04926	Relaxin signalling pathway	0.00979824	CREB5/MAP2K1/EDNRB/KRAS/PLCB4/MAPK10/ADCY3/RAF1/JUN/TGFBR2/RELA/CREB3/GNB3/PIK3CB/ATF2/CREB3L1/PLCB1/GNB2/MAPK8/NOS3/PRKACB/COL4A5/GNA15/VEGFC/ATF6B/HRAS/PIK3R3/ADCY9/MAP2K2/COL4A2/COL4A1/SMAD3/GNG2/GNAS/SRC/PIK3R1/NFKBIA/VEGFA/ADCY5/TGFB1/ARRB1/COL1A2/ARRB2/GRB2/GNG11/COL4A6/GNAI1/EDN1/SHC1/SOS1/COL1A1/SHC4/GNG5/PLCB3/NOS1/MAP2K7/PIK3CA/PRKCA/GNG12/AKT1/MAPK1/MMP9/GNB5/GNAI3/RXFP3/PRKCZ/MAPK14/ADCY2/ATF4/GNG13/COL3A1/GNB4/VEGFB/MAPK12/FOS/CREB3L4/PRKACG/GNGT2/MAPK9

### 3.3 PPI network construction and the identification of hub genes

**FIGURE 4:**
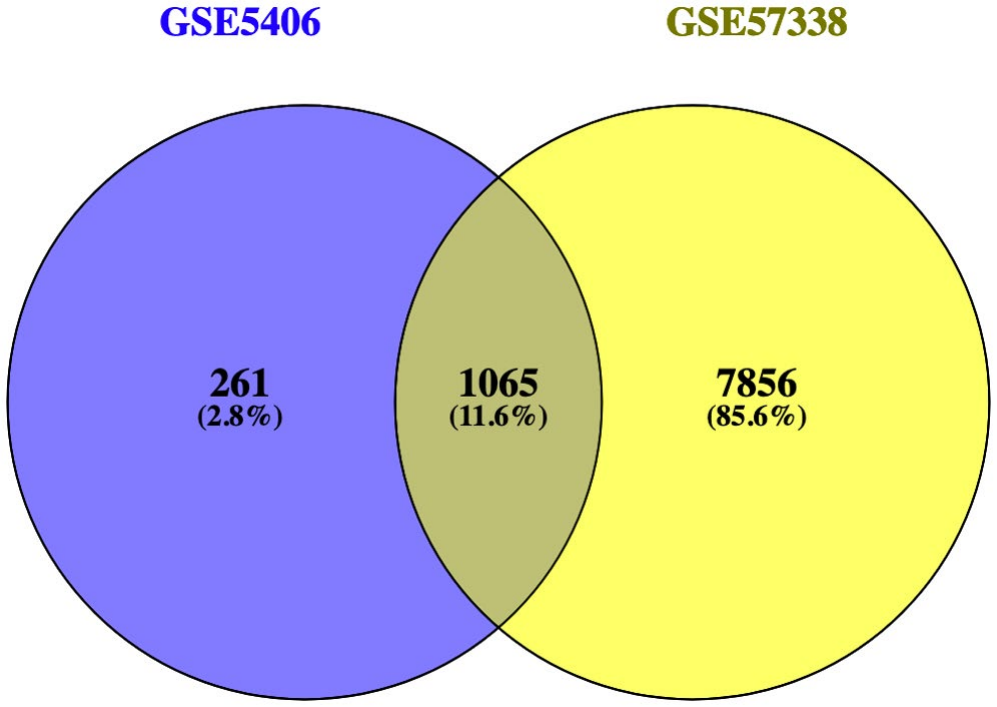
Venn diagram for the intersection of DEGs in GSE5406 and GSE57338. The genes in the cross‐set were taken out for further analysis and validation

**FIGURE 5:**
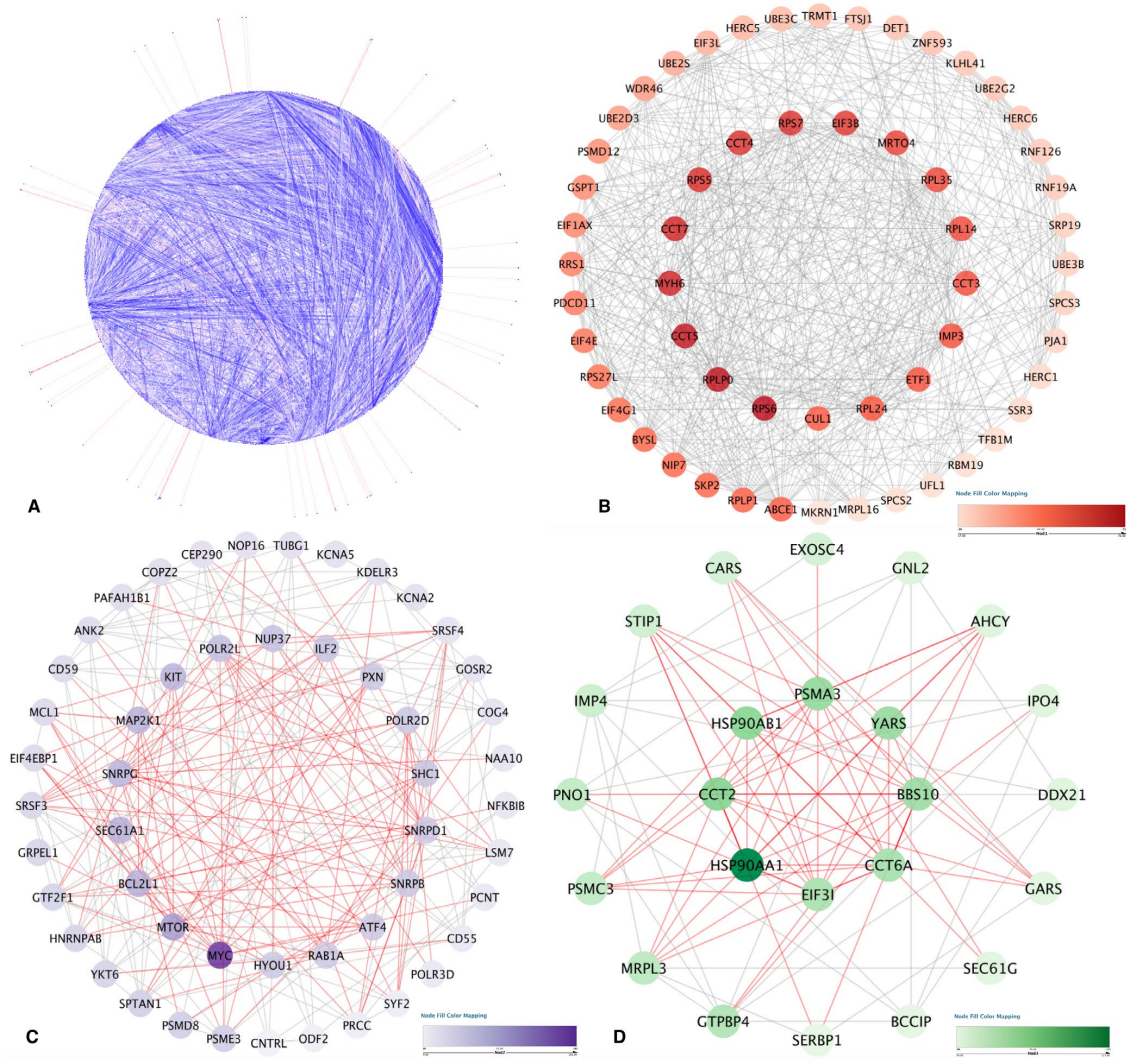
Protein‐protein interaction analysis. (A) Protein‐protein interaction network of the module genes. Each edge indicates the interaction between two genes. A colour scale was used to indicate the importance of protein nodes in the network; for example, dark red represents a high degree of importance, and light red represents a low degree importance. (B–D) The significant modules identified from the protein‐protein interaction network using the molecular complex detection method with a score of >8.0. MCODE_B_ score = 20.107, MCODE_c_ score = 18.280 and MCODE_D_ score = 8.174

There were 1065 genes duplicated in these two data sets, with adjusted *p* < 0.05 (Figure 4), and we used the STRING database to elucidate the gene‐gene interaction network of these selected genes. When the cut‐off was set as a combined score >0.9, in total of 6625 protein pairs and 868 nodes were included. Figure 5A shows the net analysis in Cytoscape. Only three modules with a score >8 were found and are shown in Figure 5B–D for detection using the MCODE app.

After integrating the above analysis, we found that only myosin heavy chain 6 (*MYH6*) could satisfy the requirement at the same time, as it was differentially expressed, was related to the ICM functional pathway and was in the MCODE‐enriched module. Next, we validated the function of *MYH6* through several different microarray data sets to further understand its relationship with ICM.

### 3.4 Hub gene validation

**FIGURE 6:**
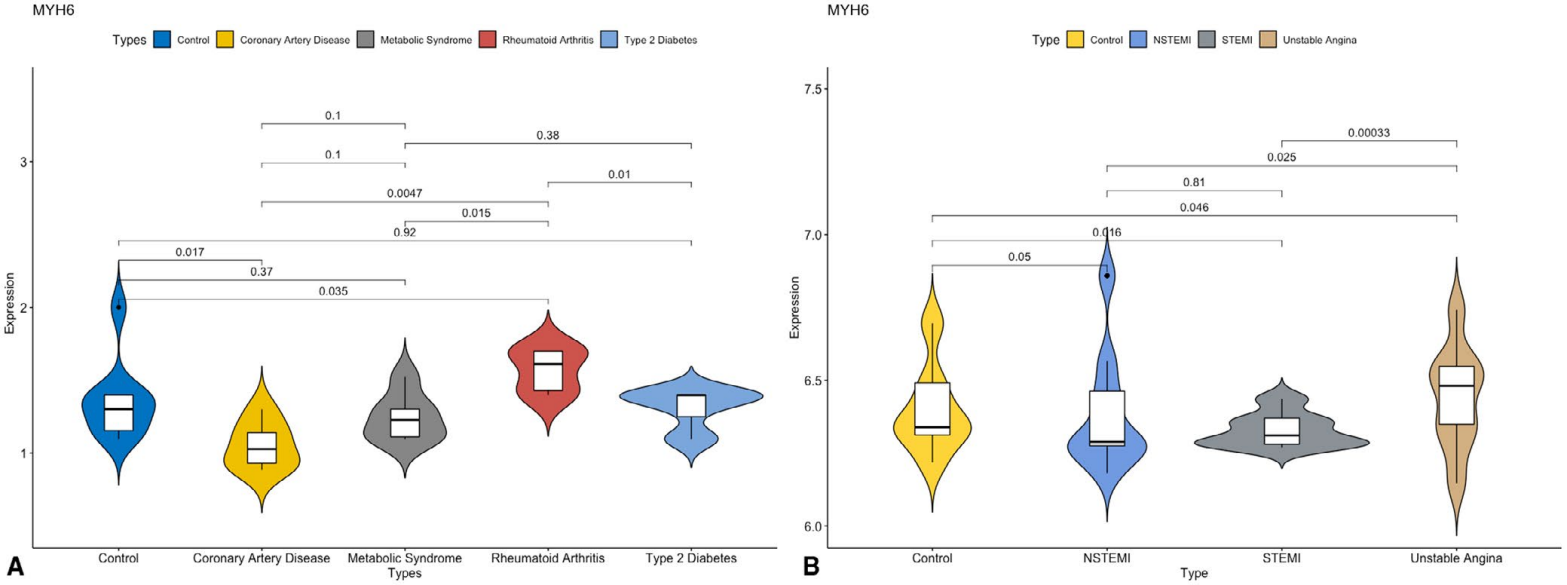
Verification of MYH6 mRNA expression levels in different datasets. (A) Expression of *MYH6* in different kinds of diseases in GSE23561; (B) Expression of *MYH6* in ACS in GSE60993.*p* < 0.05 was judged to be statistically significant within the group

First, we validated the expression of *MYH6* in GSE23561. This data set contained several ICM‐related diseases. As shown in Figure 6A, the expression of *MYH6* was lower in CAD patients than in healthy controls (*p* < 0.05). At the same time, in GSE60993, *MYH6* expression was lower in both non‐ST‐segment elevation acute myocardial infarction (NSTEMI) and ST‐segment elevation acute myocardial infarction (STEMI) patients than in healthy controls (*p* < 0.05) (Figure 6B).

**FIGURE 7:**
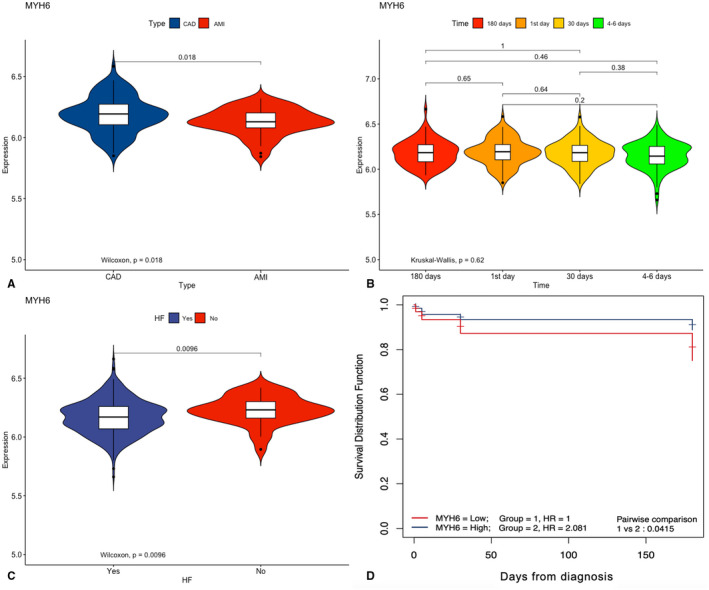
Verification of *MYH6* mRNA expression in the GSE57338 data set. (A–C) Expression of *MYH6* in different items. (D) A survival curve was drawn according to the expression level of *MYH6*, in which higher than the median was defined as high expression, and lower than the median was defined as low expression

In GSE59867, we also found that *MYH6* expression levels in AMI patients were lower than those in patients with CAD (*p* < 0.05) (Figure 7A). Subsequently, we compared the gene expression levels at different times (1 day, 4–6 days, 30 days and 180 days) after myocardial infarction. We found that there was no difference in *MYH6* expression within six months after myocardial infarction (*p* > 0.05) (Figure 7B). However, during the 180‐day follow‐up period, we found that *MYH6* expression was lower in patients with heart failure than in those without heart failure (*p* < 0.01) (Figure 7C). A survival analysis of heart failure events after myocardial infarction was performed. As shown in Figure 7D, we found that the incidence of heart failure was higher when the expression levels of *MYH6* were decreased, and this difference was statistically significant (*p* < 0.05).

## 4 DISCUSSION

The core of ischaemic cardiomyopathy is myocardial ischemia, the most common cause of which is coronary artery disease. Due to the narrowing of the coronary arteries, the blood supply to the myocardium is insufficient to meet the metabolic demands of the myocardial cells, causing myocardial necrosis, which gradually leads to myocardial fibrosis and heart enlargement, eventually progressing to heart failure.^23^, ^24^ In our current study, we found that the expression of *MYH6* was lower in CAD patients than in healthy controls, lower in AMI patients than in healthy controls, lower in CAD patients than in AMI patients and lower in HF patients than in non‐HF patients. However, there was no difference in the relative expression of *MYH6* in AMI at different periods within half a year, and the incidence of heart failure increased when *MYH6* expression was low.

Cardiac muscle myosin is a hexamer consisting of two heavy chain subunits, two light‐chain subunits and two regulatory subunits. Two types of cardiac myosin heavy chain (MyHC) isoforms have been found in humans, and the *MYH6* gene encodes the alpha heavy chain subunit of cardiac myosin (αMyHC).^25^ αMyHC is present in different amounts in mammalian hearts.^26^ Normally, αMyHC mRNA represents 20%–30% of the total myosin mRNA, and αMyHC protein represents approximately 7% of the total MyHC in mammalian nonfailing hearts. However, in the case of heart failure, the αMyHC mRNA and protein levels are downregulated to 10% and <1%, respectively. In contrast, βMyHC (encoded by *MYH7*) is upregulated.^27^, ^28^ Our results are consistent with those of previous studies. Therefore, we can speculate that the downregulation of *MYH6* expression is closely related to the occurrence of heart failure.

With the development of research technology, an increasing number of other functions of *MYH6* have been found. Carniel et al found that three heterozygous *MYH6* missense mutations were identified in dilated cardiomyopathy (DCM) probands, and a Q1065H mutation was detected in only hypertrophic cardiomyopathy (HCM) probands and was absent in 2 unaffected offspring. These results indicated that mutations in *MYH6* may cause a spectrum of phenotypes ranging from DCM to HCM.^29^ Merlo et al further found in their study that some mutations in *MYH6* caused severe adverse outcomes in DCM patients, including sudden deaths, heart failure deaths and ventricular fibrillations.^30^ Lam et al found that the *MYH6* Arg654Trp variant was a causative mutation in a family with dominantly inherited cardiac conduction disorders, and cardiac heterogeneity was also observed, including arrhythmogenic abnormalities leading to symptoms of sinus node dysfunction and sudden cardiac arrest events.^31^ These results suggested that the structural changes of the myocardium were caused by changes in *MYH6* expression and that ventricular remodelling eventually led to cardiac enlargement and sinoatrial node dysfunction, which contributed to a series of serious complications. This was also in line with our research conclusions. Myocardial infarction or severe coronary artery stenosis leads to severe myocardial ischemia and myosin necrosis,^32^ resulting in decreased expression. The clinical manifestations of ICM included ventricular remodelling, cardiac enlargement, and eventually severe heart failure.

It is important to remember that the previously analysed (GSE5406 and GSE57338) samples were derived from cardiac myocytes, while our later validation samples were derived from peripheral blood. The same conclusions could be drawn from different sources of tissue and cells, supporting the reliability of the conclusions. The expression of *MYH6* in cardiac myocytes was significantly higher than that in peripheral blood, with the same change trend. When myocardial ischaemia and necrosis occur, the expression of *MYH6* in peripheral blood also decreases.^33^ In our current study, the expression of *MYH6* in CAD patients was lower than that in the healthy control group. Therefore, whether we can predict ICM or HF through the expression level of *MYH6* in peripheral blood needs to be verified in future experiments.

This study has some shortcomings. First, our data analysis originated from different sequencing microarrays, and the results were biased due to the samples tested by these microarrays and the instruments used to test them. Although we have illustrated the role of the *MYH6* gene in the pathogenesis of ICM and HF from multiple sources of microarray data and in different dimensions, we lack validated conclusions from physical samples. Second, we identified an important role of the *MYH6* gene in the pathogenesis of ICM and HF but unfortunately failed to demonstrate the specific mechanism of this role. In future, we will conduct in vivo and in vitro experiments to verify the specific effects of this gene.

## 5 CONCLUSION

Two microarray data sets from GEO were systematically analysed. After functional enrichment and protein‐protein interaction analyses, only one gene (*MYH6*) was ultimately identified. When validated with another microarray data set, we found that the alteration in *MYH6* expression was lower in CAD patients than in healthy controls, lower in AMI patients than in healthy controls, lower in CAD patients than in AMI patients and lower in HF patients than in non‐HF patients. However, there was no difference in the relative expression of *MYH6* in AMI patients at different periods within half a year, and the incidence of heart failure increased when *MYH6* expression was low. The specific mechanism may be related to ventricular remodelling and myocardial fibrosis caused by *MYH6*, but further large‐scale experiments are needed to verify and elaborate on the specific mechanism.

## CONFLICTS OF INTEREST

The authors have no potential conflicts of interest to report.

## AUTHOR CONTRIBUTIONS

**Jian‐Hong Chen:** Conceptualization (equal); Data curation (equal); Writing‐review & editing (equal). **Lei‐Li Wang:** Formal analysis (equal); Investigation (equal). **Lin Tao:** Investigation (equal); Software (equal). **Bin Qi:** Investigation (equal); Resources (equal). **Yong Wang:** Software (equal); Validation (equal). **Yu‐Jie Guo:** Funding acquisition (equal); Project administration (equal). **Liu Miao:** Writing‐original draft (lead).

## ETHICS APPROVAL

This analysis of publicly available data does not require ethical approval.

## CONSENT FOR PUBLICATION

Not applicable.

## Supporting information

Fig S1

Table S1

Table S2

Table S3

Supplementary Material

## Data Availability

The data sets generated and/or analysed during the current study are publicly available.
