# Supplementary figures and images for: Identification of MYH6 as the potential gene for human ischaemic cardiomyopathy
Source: J Cell Mol Med. 2021 Oct 26;25(22):10736–46. doi: 10.1111/jcmm.17015 (PMC8581323; doi:10.1111/jcmm.17015)

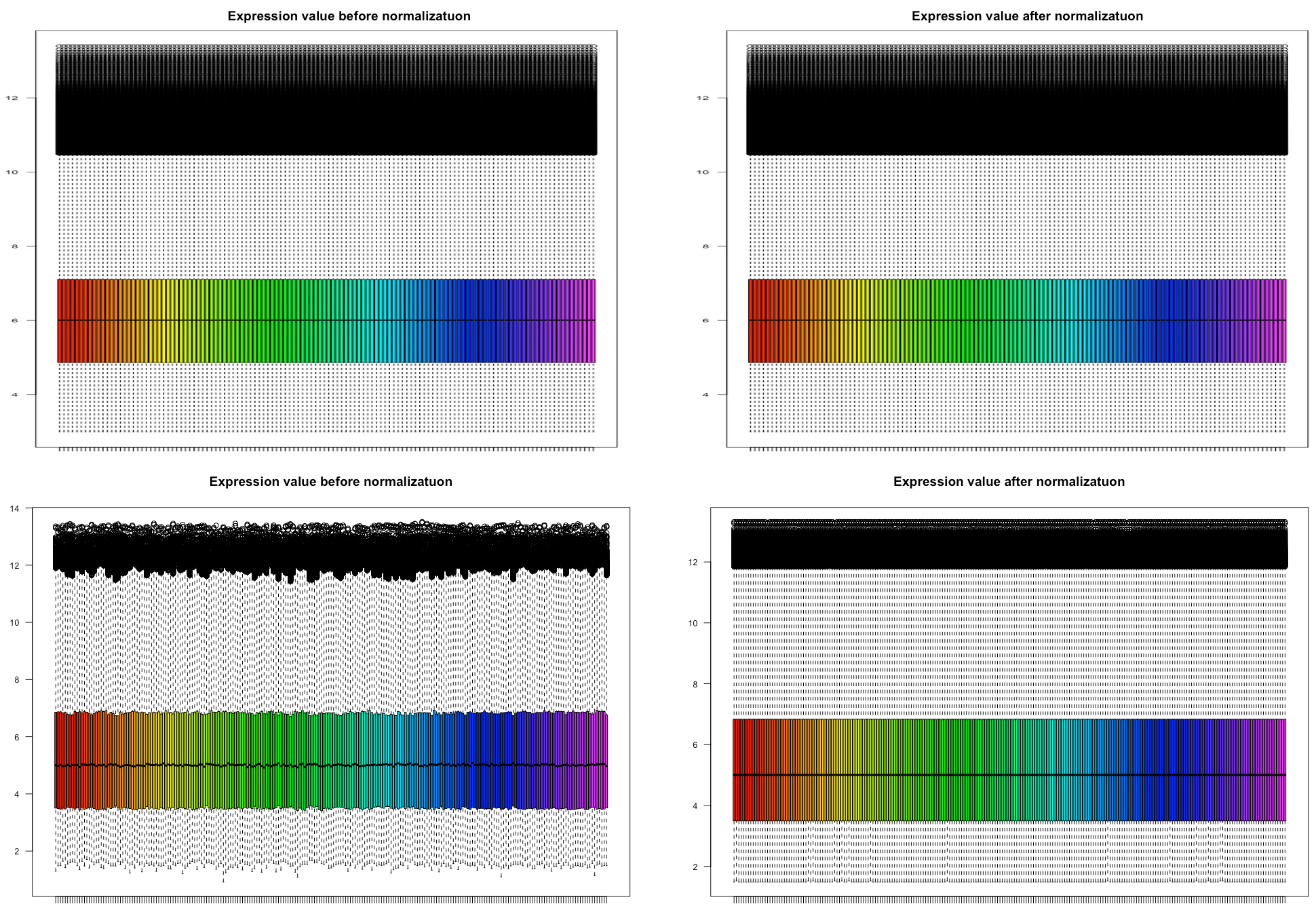


**Supplementary Figure 1: Normalized for all the samples**. On top was GSE5406 and bottom was GSE57338

Supplement: Supplementary file 1 — Fig S1 [file JCMM-25-10736-s002.docx]
