# Supplementary material for: Identification of MYH6 as the potential gene for human ischaemic cardiomyopathy
Source: J Cell Mol Med. 2021 Oct 26;25(22):10736–46. doi: 10.1111/jcmm.17015 (PMC8581323; doi:10.1111/jcmm.17015)
Supplement: Supplementary file 5 — Supplementary Material [file JCMM-25-10736-s001.docx]

**Supplementary Table 1**: GO analysis for two datasets.

**Supplementary Table 2**: KEGG analysis for two datasets.

**Supplementary Table 3**: DO analysis for two datasets.
